# Signaling pathways regulated by natural active ingredients in the fight against exercise fatigue-a review

**DOI:** 10.3389/fphar.2023.1269878

**Published:** 2023-12-14

**Authors:** Rongyue Zhao, Ruomeng Wu, Junjie Jin, Ke Ning, Zhuo Wang, Xuejie Yi, Leonid Kapilevich, Jiao Liu

**Affiliations:** ^1^ College of Exercise and Health, Shenyang Sport University, Shenyang, China; ^2^ Exercise and Health Research Center, Department of Kinesiology, Shenyang Sport University, Shenyang, Liaoning, China; ^3^ Faculty of Physical Education, Nаtionаl Reseаrch Tomsk Stаte University, Tomsk, Russia

**Keywords:** exercise fatigue, signaling pathways, natural active ingredients, recover, mechanism

## Abstract

Exercise fatigue is a normal protective mechanism of the body. However, long-term fatigue hinders normal metabolism and exercise capacity. The generation and recovery from exercise fatigue involves alterations in multiple signaling pathways, mainly AMPK, PI3K/Akt, Nrf2/ARE, NF-κB, PINK1/Parkin, and BDNF/TrkB, as well as MAPK signaling pathways that mediate energy supply, reduction of metabolites, oxidative stress homeostasis, muscle fiber type switching, and central protective effects. In recent studies, a rich variety of natural active ingredients have been identified in traditional Chinese medicines and plant extracts with anti-fatigue effects, opening up the field of research in new anti-fatigue drugs. In this review we give an overview of the signaling pathways associated with the activity of natural food active ingredients against exercise fatigue. Such a comprehensive review is necessary to understand the potential of these materials as preventive measures and treatments of exercise fatigue. We expect the findings highlighted and discussed here will help guide the development of new health products and provide a theoretical and scientific basis for future research on exercise fatigue.

## 1 Introduction

Fatigue is a complex physiological and pathological phenomenon that manifests as a feeling of exhaustion, tiredness, weariness, or lack of energy (Hsiao et al., 2018). The most common definition of fatigue is given by Chaudhuri and Behan, who define fatigue as the difficulty in starting or maintaining voluntary activity and can be categorized as peripheral and central fatigue (Liu et al., 2022). Fatigue is usually caused by intense physical work, continuous exercise, and prolonged heavy mental work, and can lead to various physical diseases, such as aging, depression, cancer, multiple sclerosis, and Parkinson’s disease (Hsiao et al., 2018; Klosterhoff et al., 2018). Exercise fatigue—which varies with intensity of exercise—is the body’s inability to maintain strength and energy output, resulting in impairment of basic energy production mechanisms and is essentially a non-pathological physiological phenomenon influenced by peripheral muscle and respiratory effects as well as the state of the central nervous system and psyche (Song et al., 2023). A certain amount of episodic fatigue—as in athletic training—can promote continuous improvements in motor skills through reasonable means of recovery; however, continuous or excessive fatigue can lead to endocrine disorders, decreased immunity, and even organic diseases that threaten physical health (Liu et al., 2022).

In recent years, researchers have found that some natural active ingredients in herbs and foods act against exercise fatigue, and the molecular mechanisms underlying these functions have become a hot research topic. The intermolecular regulatory pathways associated with exercise fatigue are complex; investigating these interactions is fundamental to understanding the onset of exercise fatigue and recovery. Accordingly, in this review, we systematically investigated the mechanisms of exercise fatigue generation and the signaling pathways mediated by natural active ingredients, resulting in fatigue recovery with the goal of deepening the knowledge base of the causes, prevention, and potential remedies of exercise fatigue.

In searching the literature in the databases PubMed, Embase, SciHub, and others, we utilized the keywords “exercise fatigue”, “signaling pathways”, “natural active ingredients”, and “recovery”. The output of this search consisted of 136 relevant studies published between 1985 and 2023.

## 2 Mechanisms of exercise fatigue generation and recovery

The following is a summary of some of the main mechanisms known to trigger exercise fatigue, as documented in recent years, along with a review of the relevant signaling pathways associated with these mechanisms.

### 2.1 Energy supply

Energy is essential for muscle contraction (Hargreaves and Spriet, 2020). Adenosine triphosphate (ATP) is the direct energy source for all vital activities in the body, and nutrients, such as phosphocreatine, glycogen, glucose, fat, and protein, provide indirect energy for exercise. During exercise, the phosphagen, lactic acid, and aerobic oxidation systems provide energy for vital activities. When the energy supply is sufficient, the muscle tissue works normally, completing the exercise process. When there is a shortage of energy, the exercise demand cannot be met, and muscle contraction is impaired, resulting in fatigue.

**FIGURE 1:**
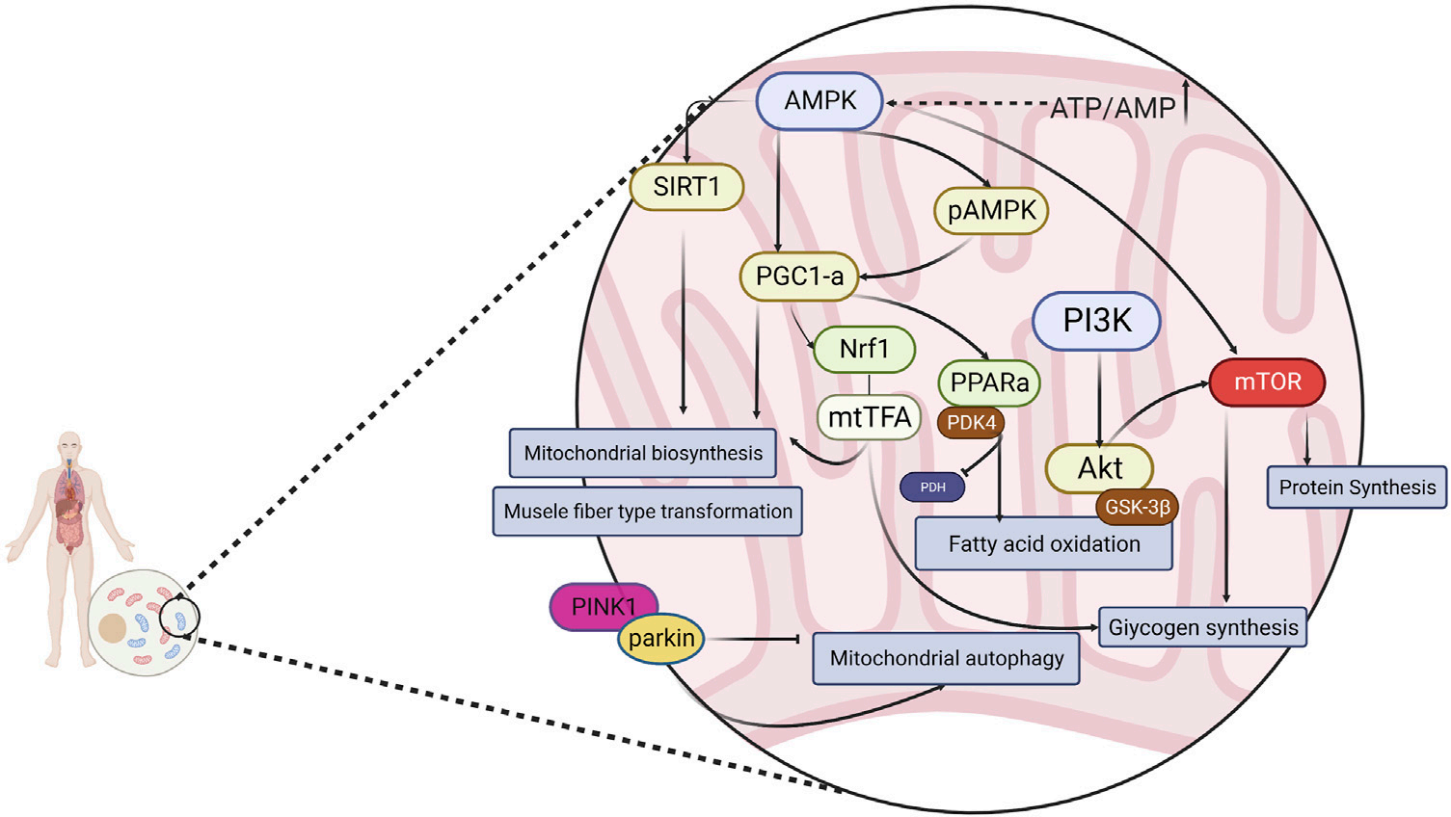
Energy metabolism is regulated through a signaling pathway consisting of AMPK and its downstream related factors.

AMP-activated protein kinase (AMPK), an “energy receptor,” is a key factor involved in the metabolism of various energy substrates and in glucose utilization, promotion of fatty acid oxidation, mitochondrial biosynthesis, and myofiber type transformation. AMPK is also involved in the regulation of cellular oxidative stress via the downstream mammalian target of rapamycin (mTOR) protein and plays a key role in the regulation of exercise fatigue. It is generally accepted that AMPK is sensitive to energy changes; in acute exercise, skeletal muscle contraction induces the AMPK signaling pathway to adapt to exercise-induced systemic metabolic responses, thereby maintaining energy homeostasis (Kjøbsted et al., 2018). As a result of elevated AMP/ATP in the cytosol, AMPK is activated and enhances glucose uptake and utilization, as well as fatty acid oxidation, producing more energy. AMPK also inhibits the glucose xenobiotic and lipid and glycogen synthesis pathways to reduce energy expenditure, thus maintaining the balance of intracellular energy metabolism. Peroxisome proliferator-activated receptor γ coactivator-1 (PGC-1α), known as the “molecular switch” for various metabolic pathways, has been shown to play an important role in slow myofibrillogenesis, regulation of glucolipid metabolism, mitochondrial production, electron transport chain, and oxygen radical elimination (Han et al., 2023; Liang et al., 2023; Yao et al., 2023). In addition, in skeletal muscle, AMPK regulates the activity of PGC-1α in the activated state, prompting the conversion of muscle fibers to type I and type IIa in muscle. Type I and type IIa muscle fibers mainly perform aerobic metabolic reactions, whereas type IId/x and type IIb muscle fibers mainly use glycolysis for energy supply. Increasing the ratio of type I and type IIa muscle fibers is conducive to improving exercise endurance (Yao et al., 2023). Silent message modifier 1 (SIRT1), which can activate the expression of the PGC-1α gene and increase the transcriptional activity of PGC-1α (thus enhancing mitochondrial biogenesis), is closely related to physiological processes, namely oxidative stress, fatty acid oxidation, and glucose synthesis (Balcerczyk and Pirola, 2010). Oxidized fatty acids are essential for the metabolic requirements of endurance exercise. Peroxisome proliferator-activated receptor (PPAR), a nuclear hormone receptor activated by fatty acids and their derivatives, acts as a lipid receptor and regulates the expression of genes related to glucose metabolism, lipids, and inflammation (Marion-Letellier et al., 2016). One of the subtypes of PPAR, PPARα, exerts a role in scavenging circulating or cellular lipids by regulating the expression of genes related to lipid metabolism in liver and skeletal muscle. By upregulating PPARα expression in skeletal muscle, fatty acid oxidation can be activated to improve exercise endurance and achieve anti-fatigue effects. Phosphatidylinositol 3-kinase (PI3K) is commonly found in somatic cells and is important for cellular energy metabolism, anti-oxidative stress, cell proliferation, and differentiation. Protein kinase B (Akt) is an important downstream molecule of PI3K, and the PI3K/Akt signaling pathway plays an important role in cell survival, metabolism, growth, differentiation, and cytoskeletal reorganization, usually by promoting glycogen synthesis in the body and exerting anti-fatigue effects (Goldar et al., 2015). PTEN-induced putative kinase 1 (PINK1) is a member of the serine/threonine protein kinase family and is highly expressed in the inner mitochondrial membrane. Parkin protein(Parkin) is a cytoplasmic E3 ubiquitin ligase that is highly expressed in skeletal muscle, brain, heart, and other tissues (Gouspillou et al., 2018). PINK1 and Parkin are key factors mediating mitochondrial autophagy during cellular removal of damaged mitochondria, and there is a direct correlation between mitochondrial autophagy and the body’s ability to resist fatigue (Botella et al., 2018). In the presence of impaired and imbalanced mitochondrial membrane potential, PINK1 translocates from the cytosol to the outer mitochondrial membrane and phosphorylates Parkin on serine 65, activating Parkin’s ligase activity and thereby regulating mitochondrial autophagy (Youle and Narendra, 2011; Zhang et al., 2019a). In summary, intervention of AMPK and downstream PGC-1α, mTOR, SIRT1, and other related factors, as well as the PINK1/Parkin, PI3K/Akt signaling pathway, can effectively alleviate exercise fatigue. (Figure 1)

### 2.2 Metabolite accumulation

The theory of metabolite accumulation suggests that athletes consume more energy substances and produce more metabolites (lactic acid, NH_4_^+^, H^+^, etc.) during high-intensity exercise than at rest. These metabolites accumulate in the blood and skeletal muscle, thereby disrupting homeostasis of the internal environment (Ament and Verkerke, 2009), which in turn causes a decrease in the motor function of muscle tissue, resulting in exercise fatigue. Lactate accumulation is considered one of the most important causes of skeletal muscle fatigue (Westerblad et al., 2002). In high-intensity exercise—as is the case with athletes—energy is mainly supplied through the lactic acid energy system: glycogen (glucose) decomposes under hypoxic conditions, producing lactic acid; with the increase in exercise intensity, the lactic acid content continues to accumulate, lactic acid dissociates, producing H^+^, which reduces the pH of the internal environment, thus inhibiting phosphorylase and phosphofructokinase activity and hindering the lactic acid energy system, resulting in insufficient supply of ATP and ensuing fatigue (Boscá et al., 1985). Research by Shanely and Coast Shanely and Coast, 2002 confirmed that the higher the lactic acid content, the more pronounced the decline in the body’s motor function and the lengthier the fatigue recovery period. During a period of strenuous exercise, the body is heavily depleted of ATP, and ammonia (NH_4_^+^) content increases in skeletal muscle. This in turn promotes glycolytic reactions, which increases blood lactic acid (BLA) and muscle lactic acid (MLA) as well as blood urea nitrogen (BUN), creatine kinase (CK), and malondialdehyde (MDA). These processes disrupts normal homeostasis and cause fatigue (Takeda et al., 2011; Gough et al., 2021).Researchers have confirmed that elevated blood ammonia levels can enter brain tissue and have neurotoxic effects on brain cells, disrupting the balance of glutamate and γ-aminobutyric acid and leading to central fatigue production (Banister and Cameron, 1990; Fernstrom and Fernstrom, 2006). Prolonged and high-intensity exercise may lead to dysfunctional ATP production and utilization, resulting in increased ATP consumption accompanied by the accumulation of metabolic byproducts such as H^+^ and inorganic phosphate (Liu et al., 2022). An increased H^+^ concentration (lower pH) leads to the inhibition of glycolysis and impaired ATP supply. H^+^ accumulation also inhibits the binding of Ga^2+^ to troponin (Tn), which affects cross-bridge circulation and sarcoplasmic reticulum Ca^2+^ pumps ultimately leading to muscle fatigue (Dutka and Lamb, 2000; Westerblad et al., 2002; Bandschapp et al., 2012; Xu et al., 2017). Clearly, the removal of metabolites is important for recovery from post-exercise fatigue; however, there is a lack of relevant studies on the molecules and mechanisms involved in this process.

### 2.3 Oxidative stress

Oxidative stress occurs as a response to harmful stimuli; excessive levels of highly reactive molecular reactive oxygen species (ROS) and reactive nitrogen species (RNS) are produced, and the degree of oxidation exceeds the rate of oxidant removal, thus causing an imbalance between the oxidant and antioxidant systems in the body, resulting in damage to large molecules, such as DNA, lipids, proteins, or even somatic tissues (De Marchi et al., 2022). Free radical generation is related to the intensity of muscle contraction. Short- or low-intensity muscle contractions induce the generation of ROS and nitric oxide (NO) at levels that increase the calcium sensitivity of myogenic fibers and enhance the skeletal muscle contractile force. In contrast, high-intensity exercise produces excessive ROS/NO, which inhibit the calcium sensitivity of myogenic fibers, resulting in impaired skeletal muscle contractile function and force output (Mason et al., 2016). In addition, the production of free radicals is related to substance transport; in a quiet state, oxidative stress in the body is low, and a moderate amount of free radicals promotes vasodilation and enhances the flow of O_2_ and nutrients. Excessive levels of free radicals, however, inhibit vasodilation and reduce blood flow, resulting in an insufficient supply of O_2_ and nutrients to body tissues and organs such as skeletal muscle, heart, skin, and brain, accelerating fatigue (Trinity et al., 2016).

**FIGURE 2:**
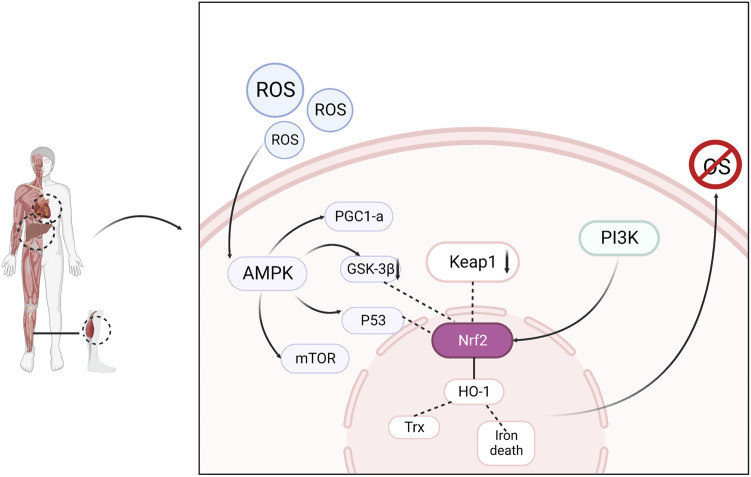
Oxidative stress is regulated through signaling pathways consisting of AMPK, Nrf2, PI3K and related downstream factors, resulting in elimination of fatigue.

Nuclear factor E2-related factor 2 (Nrf2), a major antioxidant transcription factor responsible for maintaining intracellular redox homeostasis, is expressed in most tissues. Under normal conditions, Nrf2 binds to Kelch-like ECH-associated protein 1 (Keap1) in the cytoplasm and is continuously degraded by ubiquitination. When oxidative stress occurs, Nrf2 breaks away from Keap1 and migrates to the nucleus, where it binds to the antioxidant response element (ARE) and promotes its transcription and translation, thereby producing the antioxidant enzymes HO-1 and Trx, which scavenge excess free radicals and maintain redox homeostasis (Eggler et al., 2008; Hayes and Dinkova-Kostova, 2014). AMPK activation helps reduce ROS (Swarup et al., 2008). The accumulation of ROS produced by the mitochondrial respiratory chain in skeletal muscles during exercise causes fatigue and increases oxidative damage to cells (Browning and Horton, 2004). AMPK activation prevents the overproduction and accumulation of ROS in the mitochondria (Shin and Kim, 2009). Studies have confirmed that AMPK controls exercise endurance, mitochondrial oxidative capacity, and skeletal muscle integrity (Lantier et al., 2014) and that activation of AMPK improves muscle resistance to fatigue (Wen et al., 2021). In addition to the direct activation of AMPK by elevated AMP/ATP levels, AMPK can also be activated by oxidative stress. Activated AMPK combines with downstream antioxidant genes to resist oxidative stress damage in the body (Awad et al., 2014). AMPK negatively regulates mTOR, inhibits mTOR activity, and affects the expression of downstream proteins. The AMPK/mTOR pathway is an important regulatory pathway for oxidative stress and cellular adaptation for survival. Increased p53 phosphorylation through AMPK activation stabilizes Nrf2 and upregulates antioxidant-related genes (Wu et al., 2014). The PI3K/Akt signaling pathway is one of the pathways through which the body resists damage from oxidative stress. PI3K and Akt, as key proteins in the regulation of various intracellular biological responses, can promote mitochondrial function and biosynthesis by affecting PGC1α in addition to regulating glycogen synthesis, and improve the antioxidant capacity of skeletal muscle to improve muscle atrophy and increase the endurance level of muscle exercise (Huang et al., 2023). Therefore, interventions in the Nrf2/ARE, PI3K/Akt, and AMPK/mTOR signaling pathways can effectively alleviate exercise fatigue. (Figure 2)

### 2.4 Inflammatory injury

A growing body of literature suggests that inflammation is one of the factors that induces exercise fatigue. This is because exercise injuries produce large amounts of ROS and inflammatory factors, such as interleukin-1β (IL-1β), interleukin-6 (IL-6), and tumor necrosis factor-α (TNF-α) (Liu et al., 2017; de Barcellos et al., 2021). Increased levels of ROS leads to the development of oxidative stress, which aggravates the inflammation of muscle caused by exercise, and physical function decreases as a result (Powers et al., 2011). Elevated pro-inflammatory cytokines have been reported to activate nuclear factor κ-B (NF-κB) and produce a vicious cycle of inflammatory response and mitochondrial dysfunction (Liu et al., 2022). Damaged mitochondria produce more ROS, trapping them in a vicious cycle, and leading to decreased muscle strength and fatigue (Vargas and Marino, 2014).

**FIGURE 3:**
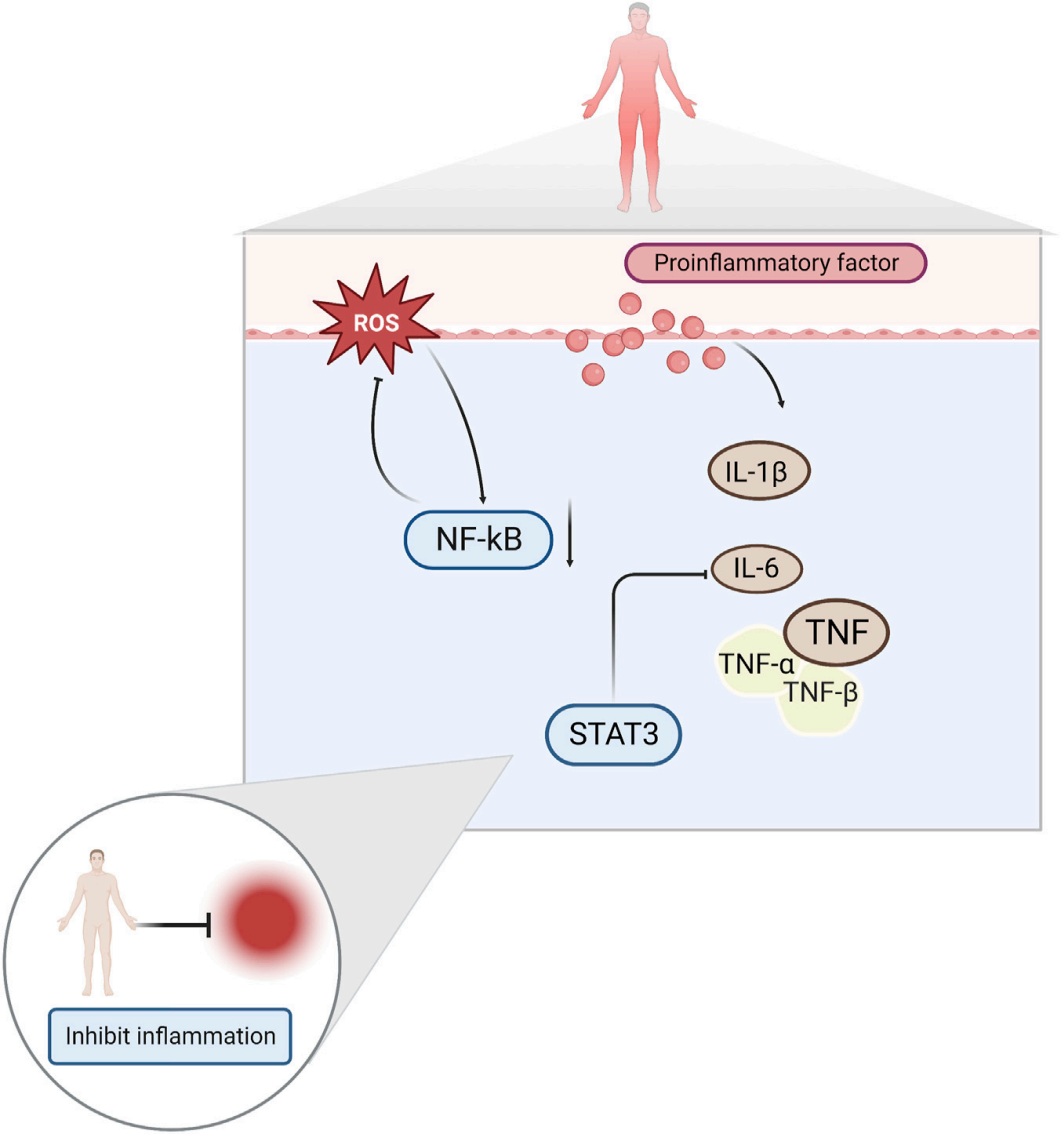
Inflammatory factors NF-κB, IL-6, TNF, and IL-1β both induce and ameliorate fatigue.

Among the proinflammatory factors released during high-intensity exercise, IL-6 significantly affects exercise fatigue (Knudsen et al., 2017). IL-6 exerts anti-inflammatory effects during moderate-to-vigorous exercise and pro-inflammatory effects during prolonged vigorous exercise (Shephard, 2002). Studies have shown that high concentrations of IL-6 are important factors that trigger fatigue in the body (Shui et al., 2019). Signal Transducer And Activator Of Transcription 3 (STAT3) is a major factor mediating IL-6 function and plays an important regulatory role in pathological processes, such as the inflammatory and immune responses (Li et al., 2020). NF-κB is a key indicator of the body’s inflammatory response. Under conditions of immune system stimulation, NF-κB migrates to the nucleus and promotes the release of various inflammatory factors, such as TNF, IL-1β and transforming growth factor-β1 (TGF-β1) (Sun et al., 2016; Fan et al., 2018). (Figure 3)

### 2.5 Protection of the central nervous system

Inhibition of the central nervous system plays an important role in exercise-induced fatigue and is considered a neurotransmitter-mediated defense (Vargas and Marino, 2014). Neural excitation of skeletal muscle contraction continuously stimulates the corresponding neurons in the cerebral cortex and maintains excitation during exercise, resulting in the continuous consumption of ATP, fatty acids, PCr, and glucose (Hirvonen et al., 1992; Hargreaves, 2015). Subsequently, the cerebral cortex and the central nervous system switch from excitation to inhibition through negative feedback regulatory mechanisms, thus preventing excessive energy consumption that would lead to fatigue (Liu et al., 2022). Well-studied brain neurotransmitters, such as serotonin (5-hydroxytryptamine, 5-HT) and dopamine (DA) have been shown to be dominant factors in the acceleration of fatigue during strenuous exercise. 5-HT is a neurotransmitter synthesized from tryptophan (TRP) and can be transported across the blood-brain barrier with the help of specific carriers (Roelands and Meeusen, 2010). Elevated concentrations of TRP in plasma and brain, subsequently leading to increased levels of 5-HT in the brain during prolonged exercise. High levels of 5-HT have been reported to promote drowsiness and perceived exertion, thereby inducing motor performance limitations and central fatigue (Meeusen et al., 2006). Heat therapy—a key limiting factor in long-term exercise—has been shown to affect core thermoregulation during exercise, which is thought to be an important fatigue-related factor (Zheng and Hasegawa, 2016). Additionally, increased 5-HT activity and decreased DA activity have been shown to contribute to exercise-induced fatigue (Meeusen et al., 2007; Leite et al., 2010).

Prolonged high-intensity exercise results in insufficient oxygen supply to the brain, increased release of oxygen free radicals and nitric oxide and can result in ischemic and hypoxic injuries, which cause apoptosis of brain tissues and decreased neurological function. The JAK kinase (Janus kinase, JAK)/signal transducers and activators of transcription (STAT) pathway regulates apoptosis caused by cerebral ischemia and ameliorates neuronal injury caused by cerebral ischemia. The JAK1/STAT pathway is an important pathway, regulating apoptosis induced by cerebral ischemia and ameliorating neuronal damage caused by cerebral ischemia (Huang et al., 2015). Extracellular signals recognize and bind to cytokine receptors on the cell membrane, form high-affinity JAK binding sites in the cytoplasm, and undergo tyrosine phosphorylation and activation; activated JAKs recruit intracytoplasmic STATs and activate hydroxytyrosine phosphorylation, which reduces the affinity of STATs for the receptor; the STATs then separate from the receptor and bind to the specific reaction of the target genes. The combination with specific response elements of the target gene induces the expression of the target gene and completes the cytokine receptor-mediated signaling process, thus exerting a variety of biological effects (Kisseleva et al., 2002).

**FIGURE 4:**
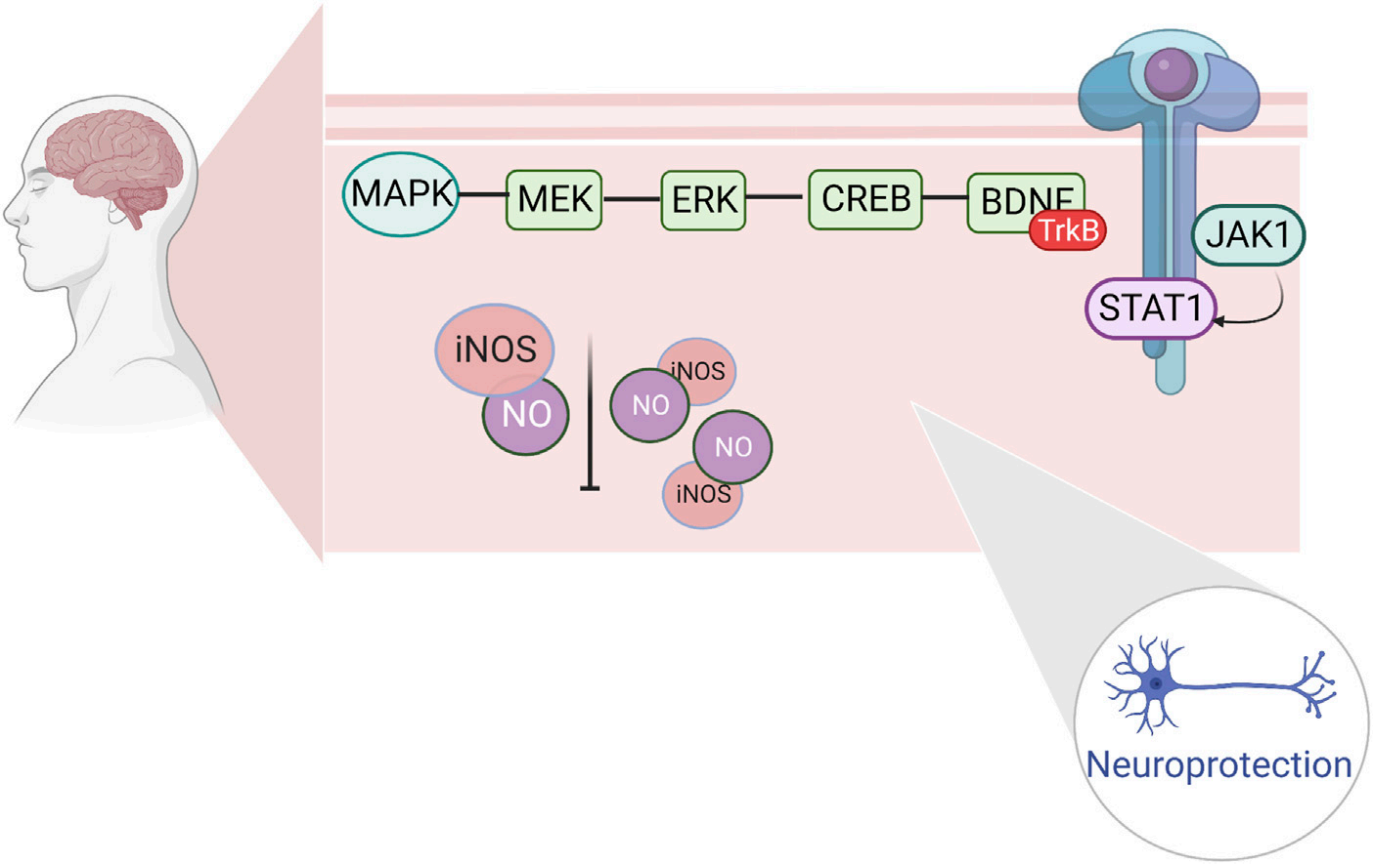
Regulation of fine apoptosis induced by cerebral ischemia by hippocampal ERK/CREB/BDNF repair and JAK1/STAT1.

Changes in neurotransmitter levels can affect the function of the central nervous system, leading to the development of exercise fatigue. One of the hot topics in recent years has been the investigation of the role of the mitogen-activated protein kinase (MAPK) signaling pathway and extracellular regulated protein kinase (ERK), a member of the MAPK family. ERK and its upstream protein, mitogen-activated protein kinase (MEK), are key components of the MAPK signaling pathway, which activates ERK molecules through phosphorylation. The activated ERK further phosphorylates downstream kinases to regulate transcription factor activity, resulting in cellular effects related to cell growth, development, and gene transcription, as well as neuronal apoptosis, and protective mechanisms (Kyriakis and Avruch, 2001). Brain-derived neurotrophic factor (BDNF), a member of the neurotrophic factor family, is highly expressed in the hippocampus of the brain (Ernfors et al., 1990). After ERK activation (cAMP-response element binding protein, CREB), phosphorylated (p)-CREB can be involved in hippocampal memory formation, thus enhancing cognitive function (Ko et al., 2018). Increased BDNF expression has a protective effect on damaged nerves and may be involved in neuronal damage in the hippocampal regions of fatigued rats (Zhu et al., 2018). The repair of hippocampal neuronal damage in rats with chronic fatigue syndrome by activating the ERK/CREB/BDNF signaling pathway suggests that this pathway may be a target for anti-motor fatigue. The electrophysiological properties of spinal cord motoneurons have been observed to change after several different forms of chronic exercise through several studies in rats. During acute exercise, rat spinal motor neurons experience a decrease in AHP amplitude and hyperpolarization of the Vth (MacDonell et al., 2015). Various forms of chronic exercise increase the excitability of rodent spinal cord motor neurons by modulating RMP, Vth, rheostat bases, discharge frequency, and AHP (Cormery et al., 2005; MacDonell et al., 2012; Krutki et al., 2015; Krutki et al., 2017; Bączyk et al., 2020). Following acute or chronic exercise, various ion channels are involved in regulating neuronal plasticity and excitability, including a) transient sodium channels, b) persistent sodium channels, c) L-type calcium channels, d) K(DR) channels, and e) K(Ca) channels (Büschges et al., 2000; Sah and Faber, 2002; Dai et al., 2018). In several studies on human motoneurons, the physiological properties of human spinal motoneurons during acute exercise were found to be similar to those of spinal motoneurons during fictive exercise in nonhuman vertebrates. Nonetheless, most studies have been based on animal models, thus limiting the conclusions that can be drawn with respect to the human central and motor systems (Forman et al., 2014; Power et al., 2022). (Figure 4)

### 2.6 Endocrine system

Strenuous exercise has been reported to activate the hypothalamus-pituitary-adrenal gland (HPA) (Ulrich-Lai and Herman, 2009; Clark and Mach, 2016), which releases catecholamines (such as epinephrine norepinephrine) and glucocorticoids into the circulatory system, resulting in elevated heart rate and blood pressure during short periods of moderate exercise. The key role of catecholamines is to regulate oxidative metabolism, lipoprotein metabolism, glycogenolysis, and energy expenditure. Therefore, elevated catecholamine concentrations lead to increased exercise capacity in the short term. However, an increase in exercise intensity and duration further leads to a decrease in energy substrates, and although catecholamine concentrations remain at high levels, a deficiency in catecholamine receptors and weakening of receptor-mediated signaling result in an inability to enhance exercise capacity through compensatory mechanisms. Thus, elevated catecholamine levels do not enhance long-term exercise capacity or even diminish it (Zouhal et al., 2008). Additionally, exercise stress increases cortisol concentrations to regulate energy, metabolic, and immune processes (Grandys et al., 2016). Prolonged and intense exercise triggers a sustained increase in cortisol levels, which suppress the HPA axis and lower serum testosterone levels, leading to decreased physical function.

## 3 Signaling pathways affected by natural active ingredients against exercise fatigue

Active ingredients in natural medicines and foods have been increasingly used in recent years for the recovery from exercise fatigue because of their better efficacy and fewer side effects. The following is a classification of the active ingredients present in medicinal foods in terms of the signaling pathways that hinder the onset of exercise fatigue or improve recovery from fatigue.

### 3.1 Polysaccharides

Polysaccharides—a class of naturally occurring biological macromolecules composed of monosaccharides—are widely found in plants, animals, and microorganisms (Shen et al., 2017). Due to their rapid absorption and metabolism, polysaccharides have been widely used in energy drinks (Boeriu, 2013). Studies have shown that the active ingredients of polysaccharides can significantly improve exercise endurance and resistance by promoting the synthesis of liver and muscle glycogen, reducing exercise metabolites, and increasing hypoxia tolerance, which are important processes in the prevention and elimination of exercise fatigue (Zhou and Jiang, 2019).

A variety of biochemical indicators can be used to assess post-exercise fatigue; *Lycium barbarum* polysaccharide (Peng et al., 2022) was found to decrease levels of MDA and BUN, and increased CAT, SOD, GSH, MG and LG levels in skeletal muscle and liver. Based on the results of RT-PCR and protein blotting, *L. barbarum* polysaccharide treatment was found to exert a significant protective effect, probably related to activation of the Nrf2/ARE signaling pathway, which attenuated the oxidative stress effect on cells by increasing the high expression of antioxidant enzymes downstream of the pathway. The effect of *L. barbarum* polysaccharide is similar to the effect of β-glucan (Salecan) in liver and skeletal muscle of fatigued rats. In addition, LBP activated AMPK/PGC-1α signaling pathway in mitochondria of rat hepatocytes and enhanced cellular energy metabolism (Xu et al., 2018). The PI3K/AKT axis regulates hepatic glycogen synthesis, gluconeogenesis, and lipid synthesis (Petersen and Shulman, 2018). Glycogen synthase kinase 3β (GSK3β) is one of the important substrates of the Akt signaling pathway, which mediates the activation of glycogen synthase and thus promotes glycogen synthesis (Feng et al., 2023). In skeletal muscle, mTOR is a common transduction signal for numerous signaling pathways and nutrients. The PI3K/Akt/mTOR signaling pathway is regulated by a variety of conditions such as growth factors, energy deficiency, insulin, hypoxia, and exercise. In addition, *Cordyceps* acidic polysaccharide modulates the PI3K/Nrf2/HO-1 pathway to exert antioxidant effects on nerve injury, suggesting that PI3K and Nrf2 combine to exert antioxidant effects (Bai et al., 2023).

### 3.2 Lignans

Lignans, a large group of natural compounds derived from the biosynthetic pathway of mangiferic acid, contain two or more basic skeletal units of phenylpropane (Scholz, 1991). Lignans are widely distributed in plants, being found in the roots, stems, leaves, flowers, fruits, seeds, and other parts (Lu and Liu, 1992; Wu et al., 2019). Recently, researchers have found that higher concentrations of lignans in flaxseed extracts protect against acute myeloid leukemia (AML) when secoisolariciresinol (SECO) released by gastrointestinal bacteria is hydroxylated and demethylated to produce mammalian lignan enterodiol (END) (Noreen et al., 2023). The structural diversity of lignans has been studied in depth, and many lignans have been found to exhibit fatigue-relieving pharmacological activities. Anwulignan (Zhang et al., 2019b), extracted from the dried ripe fruits of species of Magnoliaceae, modulates Nrf2/ARE in the mouse liver to exert anti-fatigue effects. In addition, anwulignan may exert anti-fatigue effects by upregulating p-AMPK expression, which in turn regulates both Nrf2/ARE and AMPK/PGC-1α signaling pathways. It is worth noting that AMPK not only regulates the expression of PGC-1α, but is also an important regulatory protein of Nrf2, and there are interactions between redox and metabolic signaling pathways (Zimmermann et al., 2015). Using xanthohumol (XN), a small molecule probe that activates AMPK and Nrf2, the authors determined that AMPK activation has an enhancing effect on Nrf2/hemeoxygenase1 (HO-1) signaling in mouse embryonic fibroblasts. The enhanced effect of AMPK activation on the Nrf2/HO-1 signaling pathway in mouse embryonic fibroblasts revealed, for the first time, the involvement of AMPK in the Nrf2/HO-1 signaling axis. In addition, AMPK directly phosphorylates Nrf2 and indirectly promotes the intranuclear accumulation of Nrf2 by inhibiting GSK-3β activity, and Nrf2 binding to ARE drives the expression of antioxidant enzyme genes, which in turn regulates cellular oxidative stress homeostasis (Joo et al., 2016).

One of the isoforms of PPAR, PPARδ, is involved in a variety of biological processes related to glycolipid metabolism in humans (Zhou and Jiang, 2019). Sustained activation of PPARδ increases the proportion of type I fibers in mouse skeletal muscle and modulates exercise endurance (Luquet et al., 2003). Arctigenin (Wu et al., 2014) is a lignan-like compound isolated from burdock seeds, which improves the antioxidant capacity of skeletal muscle by activating two antioxidant pathways, AMPK/PGC1α/PPARα in mitochondria and AMPK/p53/Nrf2 in the nucleus, effectively enhancing the endurance of sedentary Sprague-Dawley rats, in which AMPK can exert its biological function by regulating the downstream factor PGC-1α protein or transcriptional activity, promoting mitochondrial biosynthesis, improving mitochondrial function and energy metabolism and oxidative stress (Kim et al., 2018; Yu et al., 2018; Zhang and Liang, 2019).

### 3.3 Polyphenols

Polyphenols, such as flavonoids, tannins, and phenolic acids, are aromatic compounds containing multiple hydroxyl groups. They are secondary metabolites produced by many plants and have significant antioxidant and anti-fatigue activities, which can reduce the accumulation of free radicals and thus slow down the rate of decline in exercise capacity. In addition, polyphenolic compounds can exert their anti-fatigue effects by regulating energy substrate consumption, accumulation of metabolic byproducts, and inflammatory responses, and phenolic compounds have great potential for application in the field of anti-exercise fatigue treatments (Sharma et al., 2019).

In a previous study it was found that supplementation with >1,000 mg of polyphenols per day for 3 days or more before and after exercise promotes recovery after muscle injury through both antioxidant and anti-inflammatory mechanisms (Bowtell and Kelly, 2019). Polyphenols have been shown to inhibit cyclooxygenase activity (COX1 and COX2) in a manner similar to that of non-steroidal anti-inflammatory drugs, and there is a large amount of evidence, both *in vitro* and *in vivo*, that polyphenols have anti-inflammatory effects (Peluso et al., 2013). Many other studies have found that polyphenol supplementation before strenuous exercise reduces the markers of oxidative damage in the body after exercise (Howatson et al., 2010; Bowtell et al., 2011). The pharmacological action of turmeric is largely attributed to curcumin, a plant polyphenol with antioxidant and anti-inflammatory properties. In two clinical studies, curcumin was found to reduce muscle fatigue and soreness after exercise and to reduce, lactic acid accumulation and the level of inflammatory factors, indicating its potential to alleviate sports injuries (Mallard and Briskey, 2021; Bai et al., 2022). Curcumin upregulates Nrf2, PI3K, Akt, AMPK, and mTOR protein expression in fatigued mice, possibly by acting on PI3K/Akt/AMPK/mTOR and Nrf2 pathways, thus improving oxidative stress and energy metabolism (Chen et al., 2022c; Hu et al., 2023). Resveratrol is a natural polyphenol that exerts antifatigue effects on multiple targets, possibly by acting on core target genes, such as TP53, PIK3R1, AKT1, PIK3CA, and MAPK1 (Ma et al., 2023). Phlorizin, luteolin-6-C-neohesperidoside, and trilobati have been found to target Nrf2, reducing oxidative stress damage and inflammatory responses in liver and skeletal muscle and thus alleviating exercise fatigue (Duan et al., 2017; Ma et al., 2022; Xiao et al., 2022). Xiao et al. demonstrated for the first time that the Nrf2/ARE/iron death axis was mediated and oxidative stress induced by exercise fatigue alleviated. Iron death has been described as a newly discovered form of programmed cell death induced by iron-dependent lipid peroxidation and oxidative stress, and Nrf2 coordinates cellular antioxidant defenses in the regulation of iron death (Chen et al., 2022a). In mice, the activation of the PPAR isoform PPARδ is required for PPAR to inhibit glucose uptake and glycolytic processes and promote fat utilization, thereby enhancing exercise capacity. The authors report that this results from the upstream AMPK of PPARδ enhancing pyruvate dehydrogenase kinase 4 (PDK4) protein expression to inhibit the activity of pyruvate dehydrogenase (PDH), thus slowing down the oxidation rate of glucose and shifting the source of energy to fatty acids, which facilitates glycogen synthesis and eliminates fatigue after exercise (Fritzen et al., 2015). In mice, upregulation of PGC-1α induced by cyanidin-3-glucoside reduces blood lactate levels in gastrocnemius and biceps femoris after exercise and thus enhances exercise performance. In addition to the animal experiment, the authors of a 2017 study used C2C12 myotubes as a cellular model of skeletal muscle and found that the activation of the AMPK-PGC-1α pathway promoted mitochondrial biogenesis and exerted an anti-exercise fatigue effect (Matsukawa et al., 2017).

### 3.4 Active peptides

In recent years, bioactive peptides have become a popular research topic in the field of nutrition because of their easy absorption and multi-physiological effects. Bioactive peptides have high absorption efficiency and can participate in energy metabolism (Chai et al., 2021; Hayes, 2021). It has been reported in the literature that ginseng and walnut oligopeptides can reduce blood lactate levels and exert anti-fatigue effects in mice (Bao et al., 2016; Liu et al., 2018).

In an analysis of fermented soybean protein (Fang and Zhang, 2022), peptides were found to significantly increase myoglycogen and hepatic glycogen levels, activate p-AMPK/PGC1-α and PI3K/Akt/mTOR signaling pathways, mediate muscle protein synthesis and skeletal muscle hypertrophy, and provide energy to muscle cells, in addition to regulating the gut microbiota; however, it is still unclear what are the exact mechanisms related to the anti-fatigue effects of gut microbes. Intervention with peanut oligopeptides significantly increased the expression of mtTFA and NRF-1 in gastrocnemius mitochondria, suggesting that peanut oligopeptides improve mitochondrial function and promote energy metabolism by reducing damage to mitochondrial membranes due to oxidative stress, resulting in anti-fatigue effects (Liu et al., 2023).

### 3.5 Other bioactive components

In addition to the abovementioned components, the following active ingredients have anti-fatigue effects, such as antioxidant activity, central nervous system protection, reduction of inflammatory responses, promotion of glycogen synthesis, and regulation of intestinal microbiota responses.

Astaxanthin (Zhang and Gao, 2022), sulforaphane (Ruhee and Suzuki, 2020), gastrodin (Zhang et al., 2023), and embryo chicken egg extract (Zhang et al., 2021) enhance antioxidant stress and improve exercise tolerance in mice by interfering with the AMPK and Nrf2 signaling pathways. Extracts of *A. japonica* were found to contain dithranolone, which is the most biologically active substance in *A. japonica*. Dithranolone can inhibit the NF-κB signaling pathway and reduce the inflammatory response in skeletal muscle and cardiac muscle of fatigued mice, whereas, in the case of chronically fatigued mice, the antioxidant effect was only achieved through the AMPK/p53/Nrf pathway. Changes in AMPK phosphorylation levels and p53 expression in acutely fatigued mice were not significant, and Nrf expression decreased, suggesting that the antioxidant effect of hypericin in acutely fatigued mice may be achieved through the Nrf-related oxidative signaling pathway; however, further studies are needed (Sun et al., 2022). Supplementation with porcine whole blood protein hydrolysate also exerts anti-fatigue effects via this pathway (Jin et al., 2022), likely due to the fact that, in muscle, SIRT1 deacetylates PGC-1α and FOXO-1, leading to increased mitochondrial biosynthesis and oxidative metabolism (Ryall et al., 2015). In addition, high expression of Sirt1 promotes proliferation of myogenic cells and maintenance of muscle satellite cells (Wang et al., 2018), which are the prerequisites for enhancing sports endurance.

In addition, the MEK1-extracellular signal-regulated kinase1/2 (ERK1/2) signaling pathway produces more slow-twitch muscle fibers in the skeletal muscle, which are more fatigue-resistant than fast-twitch glycolytic fibers (Boyer et al., 2019). Lautherbach et al. found that urocortin 2 (Ucn2) may activate the ERK1/2 signaling pathway and increase muscle fatigue resistance (Lautherbach et al., 2022).

**FIGURE 5:**
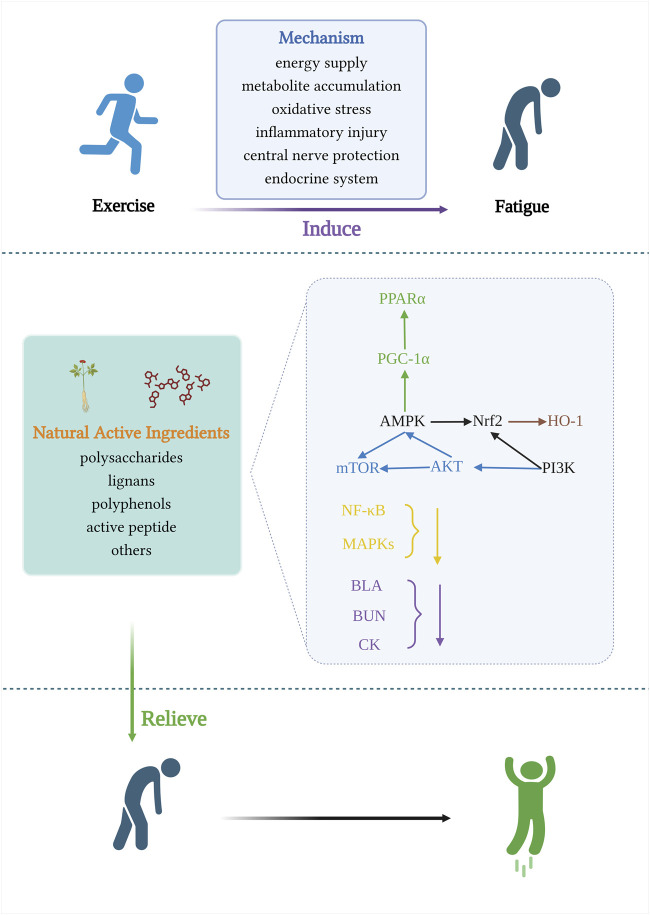
Signaling pathways involved in the mechanism of anti-exercise fatigue activity of natural food active ingredients.

High-intensity exercise is accompanied by dysbiosis of the gut microbiota, which impairs the function of the gastrointestinal tract (Miranda-Comas et al., 2022). The intestinal microbiota is a collection of microorganisms rich in metabolic enzymes, imparting a powerful metabolic capacity (Tremaroli and Bäckhed, 2012). Hydrogen is a regular product of anaerobic fermentation by intestinal flora and contributes to energy production (Pimentel et al., 2006).Luo et al. found that hydrogen water promotes glucose absorption into the bloodstream, repairs intestinal barrier damage, and improves mitochondrial biogenesis and high-intensity exercise endurance by upregulating the Pparγ/Pgc-1α/Tfam pathway (Luo et al., 2022). The use of probiotics as sports nutrition supplements is gradually increasing, and an increasing number of studies have demonstrated that supplementation with probiotics targeting the gut microbiota has great application prospects for delaying post-exercise fatigue in athletes. Its anti-fatigue performance is closely related to antioxidant capacity, reduction of lipid peroxidation and scavenging of free radicals, and the potential molecular mechanisms mainly involve activation of Nrf2 pathway, reduction of TNF-α, expression of iNOS (Kokubo et al., 2019; Cui et al., 2021; Zhang et al., 2021; Chen et al., 2022b). (Figure 5 and Table 1)

**TABLE 1:** Signal pathway of natural food active components in anti-exercise fatigue.

Mechanism of action	Ingredients	Signaling pathways
Regulation of oxidative stress in skeletal muscle, mitochondrial energy metabolism in hepatocytes	Lycium barbarum Polysaccharide	Nrf2/ARE,AMPK/PGC-1α
Anti-oxidation	β-glucan (Salecan)	Nrf2/HO-1/Trx
Anti-oxidative nerve damage	Cordyceps acidi polysaccharide	PI3K/NRF2/HO-1
Regulation of mitochondrial energy metabolism, antioxidant	Anwulignan	AMPK/PGC-1α, Nrf2/ARE
Improves energy metabolism and antioxidant capacity of skeletal muscle	Arctigenin	AMPK/PGC-1α/PPARα, AMPK/p53/Nrf2
Improves oxidative stress and energy metabolism in the body	Curcumin	PI3K/Akt/AMPK/mTOR, Nrf2/ARE
Reduction of oxidative stress damage	Phlorizin	Nrf2/ARE
	Luteolin-6-C-Neohesperidoside	Nrf2/ARE
	Gastrodin	
	Sulforaphane	
	Astaxanthin	
	Trilobati	Nrf2/ARE/Iron Death
	Embryo chicken egg extract	AMPK/mTOR
Mediates protein synthesis and skeletal muscle hypertrophy	Fermented soybean protein peptides	pAMPK/PGC-1αPI3K/Akt/mTOR
Improves damaged myocardial function, antioxidants and inflammation	hypericin	BDNF/TrkB, AMPK/p53/Nrf, NF-κB
Promotes slow muscle fiber expression and mitochondrial function	Porcine Whole Blood Protein Hydrolysate	AMPK/SIRT1
Promotes slow muscle fiber production	Urocortin 2	ERK1/2
Regulation of mitochondrial biogenesis	Hydrogen water	Pparγ/Pgc-1α/Tfam

## 4 Conclusion and outlook

Exercise fatigue is a complex physiological and biochemical process that affects the entire body. Exercise fatigue involves mechanisms such as energy depletion, metabolite accumulation, oxidative stress, and inflammation, and is also closely related to neurotransmitters and the endocrine system. As for the molecular mechanisms involved in the development and recovery of exercise fatigue, most of these are limited to a series of intermolecular changes resulting from the intervention of edible ingredients; far less research has been conducted on the intervention of the central system and gut microorganisms in key signaling pathways. The application of Chinese medicine in the recovery of exercise fatigue has been receiving increasing attention given that such treatments are relatively safe, have fewer side effects, and can be used to improve sports endurance and accelerate the recovery of exercise fatigue by adding or replacing single medicine components on the basis of the original formula.

The mechanisms of the existing signaling pathways involved in the elimination of exercise fatigue are not well understood and need to be further explored. In this review, focused on the physiological mechanism of exercise fatigue, we collected relevant data and systematically sorted out the signaling pathways related to the production and recovery of exercise fatigue from the molecular level, and the interactions between the molecules involved cross each other to form a complex signaling pathway “network,” and the interactions between these molecules are at the root of the occurrence and recovery of exercise fatigue. Moreover, the active extracts of drugs and foods regulate physiological and biochemical processes in the body through relevant pathways to prevent or alleviate exercise fatigue. Among them, the target protein AMPK is activated to mutate and stimulate PGC-1α, PPARα, and mTOR transduction pathways to enhance the utilization of fatty acids and glucose by peripheral tissues, especially skeletal muscle, mediating recovery from exercise fatigue from signaling pathways related to regulation of energy levels, metabolic substrate utilization, adaptation of cellular OS, mitochondrial biosynthesis, and changes in muscle fiber type. pi3k/Akt are mainly involved in promoting glycogen synthesis in the body. nrf2/ARE regulates cellular oxidative stress homeostasis. pink1/parkin mediates mitochondrial autophagy. nf-κb signaling pathway attenuates inflammatory damage. MAPK, downstream ERK, and MEK regulate the oxygen radical-scavenging capacity of skeletal muscles and promote slow muscle fiber production. MEK/ERK and BDNF/TrkB are involved in repairing hippocampal injury. In skeletal muscle, mTOR is a co-transduction signal for numerous signaling pathways and nutrients. For example, AMPK/mTOR is an important regulatory pathway for oxidative stress and cellular adaptation for survival. PI3K/Akt/mTOR mediates muscle protein synthesis and participates in skeletal muscle hypertrophy by providing energy to muscle cells. In addition, regulation of microbiota may open new avenues for the amelioration of diseases caused by excessive exercise and provide a basis for the prevention and treatment of exercise fatigue, but there is a lack in the understanding of the supporting molecular mechanisms.

## 5 Recommendations

Because the occurrence of exercise fatigue involves a wide range of mechanisms, if there are omissions in the signaling pathways, further additions are required to repair the mechanism. There is a lack of effective cellular experiments and further studies should be conducted at both the cellular and molecular levels. At present, most studies on fatigue recovery are performed with animal exhaustion exercises as the model, and the modeling methods include swimming, stage running, stick turning, and pole climbing experiments, which depend on subjective judgment and have certain limitations. Existing anti-fatigue animal models are mostly based on physiological fatigue, ignoring psychological fatigue. Most research hotspots on exercise fatigue are based on experimental studies that lack clinical application, and clinical trials should be conducted.
